# Recombinant ACE2 Expression Is Required for SARS-CoV-2 To Infect Primary Human Endothelial Cells and Induce Inflammatory and Procoagulative Responses

**DOI:** 10.1128/mBio.03185-20

**Published:** 2020-12-11

**Authors:** Jonas Nascimento Conde, William R. Schutt, Elena E. Gorbunova, Erich R. Mackow

**Affiliations:** a Department of Microbiology and Immunology, Stony Brook University, Stony Brook, New York, USA; Indiana University Bloomington

**Keywords:** ACE2, COVID-19, SARS-CoV-2, coagulopathy, endothelial cells, endotheliitis, inflammation, pulmonary

## Abstract

SARS-CoV-2 causes COVID-19, an acute respiratory distress syndrome (ARDS) characterized by pulmonary edema, viral pneumonia, multiorgan dysfunction, coagulopathy, and inflammation. SARS-CoV-2 uses angiotensin-converting enzyme 2 (ACE2) receptors to infect and damage ciliated epithelial cells in the upper respiratory tract. In alveoli, gas exchange occurs across an epithelial-endothelial barrier that ties respiration to endothelial cell (EC) regulation of edema, coagulation, and inflammation. How SARS-CoV-2 dysregulates vascular functions to cause ARDS in COVID-19 patients remains an enigma focused on dysregulated EC responses. Whether SARS-CoV-2 directly or indirectly affects functions of the endothelium remains to be resolved and is critical to understanding SARS-CoV-2 pathogenesis and therapeutic targets. We demonstrate that primary human ECs lack ACE2 receptors at protein and RNA levels and that SARS-CoV-2 is incapable of directly infecting ECs derived from pulmonary, cardiac, brain, umbilical vein, or kidney tissues. In contrast, pulmonary ECs transduced with recombinant ACE2 receptors are infected by SARS-CoV-2 and result in high viral titers (∼1 × 10^7^/ml), multinucleate syncytia, and EC lysis. SARS-CoV-2 infection of ACE2-expressing ECs elicits procoagulative and inflammatory responses observed in COVID-19 patients. The inability of SARS-CoV-2 to directly infect and lyse ECs without ACE2 expression explains the lack of vascular hemorrhage in COVID-19 patients and indicates that the endothelium is not a primary target of SARS-CoV-2 infection. These findings are consistent with SARS-CoV-2 indirectly activating EC programs that regulate thrombosis and endotheliitis in COVID-19 patients and focus strategies on therapeutically targeting epithelial and inflammatory responses that activate the endothelium or initiate limited ACE2-independent EC infection.

## Abstract (precis)

SARS-CoV-2 infects pulmonary epithelial cells through ACE2 receptors and causes ARDS. COVID-19 causes progressive respiratory failure resulting from diffuse alveolar damage and systemic coagulopathy, thrombosis, and capillary inflammation that tie alveolar responses to EC dysfunction.

## OBSERVATION

SARS-CoV-2 predominantly infects the epithelium of upper and lower airways causing pulmonary pathology and acute respiratory distress syndrome (ARDS) (1). COVID-19 is characterized by progressive respiratory failure resulting from diffuse alveolar damage, inflammatory infiltrates, endotheliitis, and pulmonary and systemic coagulopathy forming obstructive microthrombi with multiorgan dysfunction (1–3). Collectively, these findings indicate that initial pulmonary epithelial infection leads to COVID-19 vasculopathy with featured alveolar endothelial cell (EC) dysfunction playing a key role in anomalous vascular leakage, coagulation, and inflammation. In COVID-19 patients, procoagulative responses are associated with altered von Willebrand factor (vWF) and thrombomodulin expression and the induction of proinflammatory cytokines (interleukin-1 [IL-1], IL-6, tumor necrosis factor alpha [TNF-α]) that further implicate activation of the endothelium in myocarditis and vasculopathy (1–4).

Despite coagulopathy and capillary inflammation in COVID-19 patients, it is unclear whether ECs are directly infected by SARS-CoV-2 or whether EC activation is an indirect response to primary alveolar epithelial cell damage and inflammatory responses (1–3). SARS-CoV-2 infects cells by attaching to human angiotensin-converting enzyme 2 (ACE2) receptors (5–7). Rationales for SARS-CoV-2 directly infecting ECs originated from prothrombotic findings, endotheliitis, protective ACE2 functions, and reports that ECs express cellular ACE2 receptors (8–10). However, several studies demonstrate that in the vasculature ACE2 is confined to the tunica media, colocalizing with smooth muscle actin, not the endothelium (11–14). CDC analysis of COVID-19 patient tissues indicates that SARS-CoV-2 is detectable in airways, pneumocytes, alveolar macrophages, and lymph nodes but not in ECs or other extrapulmonary tissues (1). In retrospect, there are minimal data supporting SARS-CoV-2 infection of ECs and no immunohistochemical studies demonstrating the colocalization of SARS-CoV-2 antigens with EC markers in pulmonary or renal tissues, which express ACE2 on adjacent epithelial cells. Nearly all studies reference electron microscopy data displaying two potential SARS-CoV-2 particles (3, 15), which instead of virus have been implicated as being endoplasmic reticulum (ER) vesicles (16).

**FIG 1:**
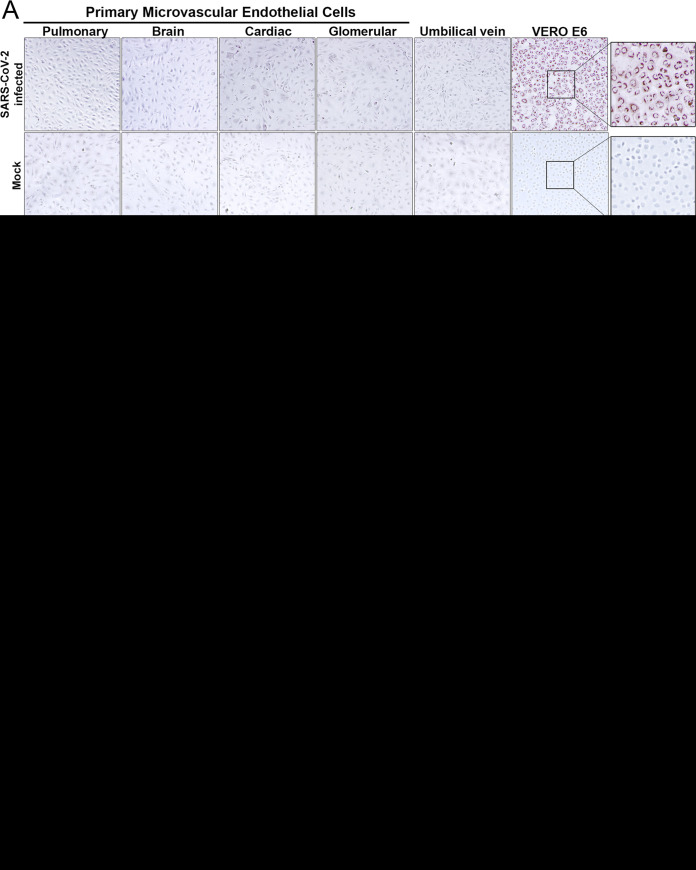
SARS-CoV-2 fails to infect primary human endothelial cells without rACE2 expression. (A) Primary human microvascular endothelial cells from pulmonary (hPMECs), brain (hBMECs), cardiac (hCMECs), or glomerular (hGMECs) tissue or umbilical vein (HUVECs) or VeroE6 cells were mock or SARS-CoV-2 (strain WA) infected (multiplicity of infection [MOI] of 10) and at 24 hpi immunoperoxidase stained for nucleocapsid protein. (B and C) Primary human ECs and VeroE6, HEK293T, and Calu3 cells were analyzed by qRT-PCR for ACE2 mRNA (B) and by Western blotting for expressed ACE2 (C). HUVECs contain a potential ACE2 truncation lacking the N-terminal SARS-CoV-2 binding domain. GAPDH, glyceraldehyde-3-phosphate dehydrogenase. (D) Primary human pulmonary ECs lentivirus transduced to express recombinant ACE2 were infected with SARS-CoV-2 (MOI of 1) for 6 to 48 hpi. (E) ACE2-hPMECs or wild-type (WT)-hPMECs were immunoperoxidase stained for nucleocapsid protein. hPMECs or rACE2-hPMECs were analyzed by immunofluorescence assay (IFA) for the EC marker PECAM-1 and ACE2. (F) Following SARS-CoV-2 infection, rACE2-hPMECs were analyzed by IFA for coexpressed ACE2 and nucleocapsid protein (N) or PECAM-1 expression. Bars represent 50 μm. (G) For supernatants of SARS-CoV-2-infected (MOI of 1) WT hPMECs, hBMECs, rACE2-hPMECs, rACE2-hBMECs, and VeroE6 cells, titers were determined 2 to 72 hpi (limit of detection, <10 focus-forming units [FFU]/ml).

Nonetheless, pathological findings in COVID-19 patients demonstrate the dysregulation of EC functions (17); however, the mechanism(s) of endothelial damage and activation in SARS-CoV-2-directed coagulopathy and inflammation remains to be revealed (2, 4). Our initial studies were predicated on ACE2 receptors directing SARS-CoV-2 infection and dysregulation of normal EC functions. We critically analyzed SARS-CoV-2 infection of primary human ECs derived from lung, heart, kidney, brain, and umbilical veins (see Text S1 in the supplemental material). Remarkably, we found that SARS-CoV-2 failed to infect primary human ECs derived from any organ. In contrast to the complete infection of VeroE6 cells, no SARS-CoV-2-infected ECs were detected, by N or Spike antigen immunostaining, at any multiplicity of infection or plating cell density (Fig. 1A). Consistent with this, both ACE2 RNA and protein, found in VeroE6 and Calu3 cells, were undetectable in ECs (Fig. 1B and C), and no viral progeny was detected in the supernatants of SARS-CoV-2-infected human ECs (1 to 3 days postinfection [dpi]) (Fig. 1G).

10.1128/mBio.03185-20.1 TEXT S1 The methods used within the studies describing cells, virus, SARS-CoV-2 infection, ACE2 lentivirus transduction, qRT-PCR analysis, Western blotting, confocal immunofluorescence, and statistical analysis are presented. Download Text S1, DOCX file, 0.1 MB. Copyright © 2020 Nascimento Conde et al. 2020 Nascimento Conde et al. This content is distributed under the terms of the Creative Commons Attribution 4.0 International license.

To determine whether SARS-CoV-2 infection of ECs is receptor restricted, we lentivirus transduced primary human pulmonary and brain ECs to express ACE2 and evaluated viral replication and protein expression. We found that expressing ACE2 in primary human ECs permitted SARS-CoV-2 to ubiquitously and productively infect ECs, reaching viral titers of 1 × 10^7^ to 3 × 10^7^ (1 to 3 dpi) (Fig. 1D and G) (Text S1). SARS-CoV-2 infection colocalized with ACE2-expressing ECs (Fig. 1E and F) and resulted in detectable N protein at 4 to 6 h postinfection (hpi) and multinucleate syncytia and EC lysis at 12 to 24 hpi (Fig. 1D and F). Collectively, these findings demonstrate that primary human ECs lack ACE2 receptors required for SARS-CoV-2 infection but express proteases essential for SARS-CoV-2 infection. These findings suggest that SARS-CoV-2 may cause procoagulative endotheliitis through indirect EC dysregulation mechanisms or as a result of ACE2-independent, or induction-directed, infection of a small number of activated ECs.

**FIG 2:**
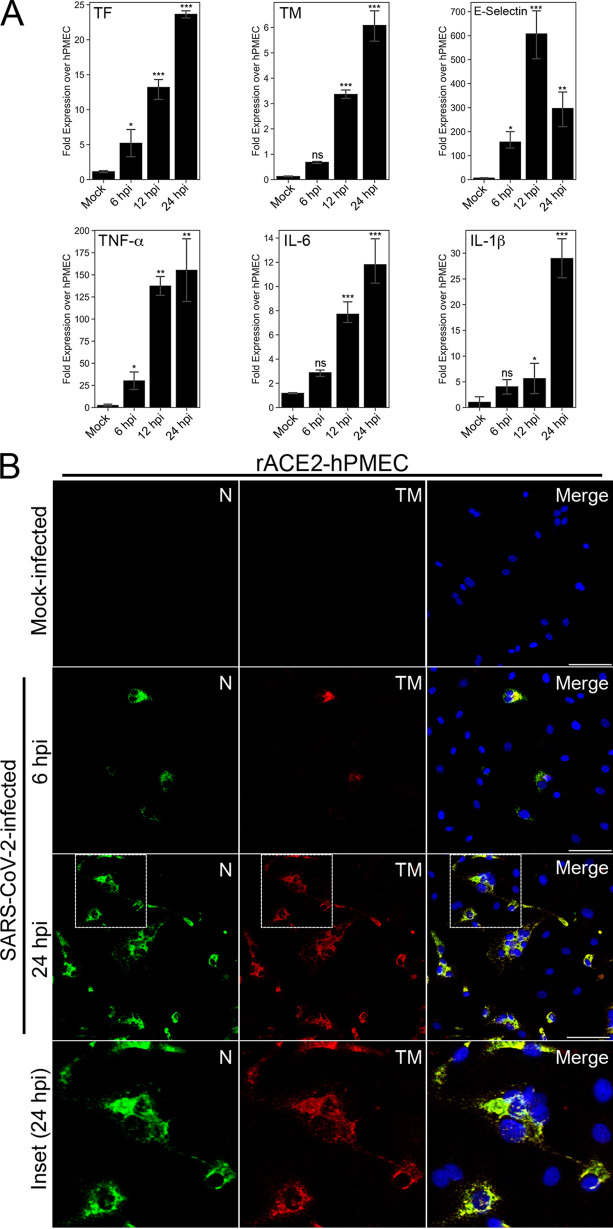
Recombinant ACE2-expressing ECs elicit procoagulation and inflammatory responses. hPMECs expressing recombinant ACE2 (hPMEC-rACE2) were synchronously infected with SARS-CoV-2 and analyzed by qRT-PCR for changes in the mRNA levels of coagulation and inflammatory responses 6 to 24 hpi. (A) Levels of tissue factor (TF), thrombomodulin (TM), tumor necrosis factor alpha (TNFα), interleukin 6 (IL-6), IL-1β, and E-selectin were found to increase dramatically in SARS-CoV-2-infected ECs. (B) The induction of TM in SARS-CoV-2-infected rACE2-hPMECs (MOI of 1) was monitored by IFA of viral nucleocapsid (N) and cellularly expressed thrombomodulin (TM) from 6 to 24 hpi.

The potential for damage, inflammation, or activation to conditionally permit SARS-CoV-2 infection of a small EC subset (12, 18, 19) prompted us to analyze cellular responses that may contribute to COVID-19 pathogenesis. We analyzed transcriptional responses of ACE2-expressing ECs to SARS-CoV-2 infection and found significant changes in mRNAs that regulate coagulation and inflammation from 6 to 24 h (Text S1) including 2- to 3-fold decreases in PAI-1, antithrombin, and factor VIII and increases in tissue factor (24-fold), thrombomodulin (TM) (6-fold), vWF (3-fold), thrombin receptors (PAR1/3, 3-fold), EGR-1 (37-fold), E-selectin (600-fold), IL-1β (28-fold), IL-6 (12-fold), and TNF-α (160-fold) (20, 21) (Fig. 2A). SARS-CoV-2 selectively induced thrombomodulin in infected recombinant ACE2-expressing human microvascular endothelial cells from pulmonary tissue (rACE2-hPMECs), with TM internally colocalized with viral N protein (Fig. 2B), suggesting the potential for SARS-CoV-2 to sequester a coagulation-inhibiting EC surface receptor (20). However, a comprehensive assessment of coagulation and inflammatory mediators in SARS-CoV-2-infected epithelial and endothelial cells is required to fully understand EC activation events and complex coagulation factor and inflammatory responses that can be therapeutically targeted.

Our findings indicate that the absence of ACE2 prevents SARS-CoV-2 infection of human ECs and suggests that ECs are not primary targets of SARS-CoV-2 infection in COVID-19 patients. Consistent with this, COVID-19 does not result in Ebola-like hemorrhagic disease that would likely result from lytic SARS-CoV-2 infection of ACE2-expressing ECs. The inability of SARS-CoV-2 to infect human ECs is supported by low ACE2 expression in the highly vascularized lower respiratory tract (22), CDC and primary human EC infection findings (1, 14, 22), and the presence of ACE2 in vascular smooth muscle and heart muscle cells (11, 18, 23, 24) but not the EC lining of vessels (12–14, 23). These findings support a secondary role of the endothelium, perhaps in response to epithelial cell damage and cross talk, alveolar tissue factor/basement membrane exposure, or inflammatory EC activation, that directs a coagulative, endotheliitic state (1, 3, 17, 25).

Our findings do not address whether SARS-CoV-2 infection of pulmonary epithelial cells permits SARS-CoV-2 to selectively infect or activate ECs. In the course of these experiments, we tested, but were unable to define, conditions that permitted SARS-CoV-2 infection of pulmonary ECs (addition of angiotensin II (AngII), activating AMP kinase, hypoxia, TNF-α, IL-1β, IL-6, bradykinin, or endothelin-1). However, it remains conceivable that COVID-19 epithelial cell or immune cell responses selectively activate the endothelium (2) and permit a subset of ECs to be infected over time (19). Reported EC heterogeneity in response to acute lung injury (19) supports the potential for infection of a subset of ECs, and one report suggests that 1/250 ECs are ACE2 positive and that both SARS-CoV-2 and influenza virus increase the number of ACE2-positive ECs (3). Yet SARS-CoV-2 infection of ACE2-expressing ECs remains to be demonstrated in COVID-19 patients and is at odds with current findings and additional studies indicating that ECs lack ACE2 (12–14, 23).

Consistent with COVID-19 disease, we found that SARS-CoV-2 infection of ECs induces procoagulative and inflammatory mediators (1–3, 17, 21). Our finding that the coagulation initiator tissue factor is highly induced, whereas the coagulation inhibitor thrombomodulin is induced and may be sequestered within ECs, provides potential thrombotic mechanisms, while findings of induced cytokines and E-selectin are consistent with inflammation and endotheliitis (3, 20, 25, 26). These results rationalize a detailed analysis of EC-expressed procoagulative and inflammatory factors and the potential role of targeting thrombomodulin, TNF-α, and E-selectin in resolving EC-directed COVID-19 coagulation and inflammation (3, 4, 20, 26). However, in the absence of EC infection, damage to alveolar epithelial cells may alone initiate coagulopathy through tissue factor, intra-alveolar fibrin deposition, and common EC basement membrane exposure that triggers activation of the endothelium (25, 27). In COVID-19 patients, EC damage and activation responses are also likely to be exacerbated by impaired ACE2 activity that increases the severity of ARDS, AngII-directed EC damage, bradykinin-directed permeability and inflammation, and the loss of protective anti-inflammatory Ang1-7 responses (9, 24, 28–30). Overall, our data suggest that SARS-CoV-2 is likely to indirectly dysregulate EC functions, and this explains the absence of an acute lytic infection of ECs and the chronic vascular disease process that over time evolves into an aberrant prothrombotic endotheliitis in COVID-19 patients. These findings focus strategies on therapeutically targeting epithelial and inflammatory responses that activate the endothelium or initiate limited ACE2-independent EC infection.
