# Supplementary material for: Recombinant ACE2 Expression Is Required for SARS-CoV-2 To Infect Primary Human Endothelial Cells and Induce Inflammatory and Procoagulative Responses
Source: mBio. 2020 Dec 11;11(6):e03185-20. doi: 10.1128/mBio.03185-20 (PMC7751258; doi:10.1128/mBio.03185-20)
Supplement: TEXT S1 [file mBio.03185-20-s0001.docx]

**Supplemental Material**

**S1-Methods**

**Cells and virus.** Primary human microvascular endothelial cells derived from pulmonary (hPMECs, H-6011), brain (hBMECs; H-6023), cardiac (hCMECs; H-6024), and glomerular (hGMECs; H-6014G) tissues were purchased from Cell Biologics, and human umbilical vein ECs (HUVECs; C-2519) were purchased from Lonza. Primary human ECs were grown in endothelial cell basal medium-2 (EBM-2; Lonza) supplemented with EGM-2 MV SingleQuots (Lonza) and incubated at 37°C and 5% CO_2_. HEK293T, VeroE6 and Calu-3 cells (ATCC) were grown in DMEM (Dulbecco’s modified Eagle’s medium) supplemented with 8% fetal bovine serum (FBS) and penicillin (100 µg/ml), streptomycin sulfate (100 µg/ ml), and amphotericin B (50 µg/ml; Mediatech) at 37°C and 5% CO_2_. SARS-CoV-2 (USA-WA1/2020) was obtained from BEI Resources (NR-52281), (at multiplicity of infection [MOI] of 0.1), and propagated for 2 days in VeroE6 cells in DMEM with 2% FBS.

**ACE2 Lentivirus Transduction.** Human ACE2 was PCR amplified from hACE2 Addgene plasmid (#1786) (forward, AAACGTCTCGGATCCATGTCAAGCTCTTCCTGG, reverse AAACGTCTCTCTAGACTAAAAGGAGGTCTGAACATCATC) and subcloned into Bam-HI-XbaI sites of pLenti-Puro plasmid. Lentivirus vectors were generated by polyethylenimine (PEI) transfection of HEK293T cells. HEK293T cells (3.8 x 10^6^) were transfected with pLenti-ACE2 expression plasmid in a 3:2:1 ratio with psPAX2 and pCMV-VSV-G (1:3 DNA/PEI ratio). After 18h, media was replaced and Lentivirus containing supernatants were harvested 72 h post-transduction and 0.45-µm PVDF filtered. Lentivirus (plent-ACE2) stocks were transduced into primary hPMECs and hBMECs, puromycin selected (1 µg/ml) for 72 h and analyzed for ACE2 expression by Western blot and immunostaining using antibody to ACE2 (ACE2-10108-T24, Sino Biological).

**SARS-CoV-2 infection assays.** SARS-CoV-2 at different MOIs (passage 3 0.1-100) was adsorbed to hPMEC, hBMEC, hCMEC, hGMEC, HUVEC and VeroE6 monolayers plated at different cell densities (40-100%) for 1-16 hours. Following adsorption, monolayers were washed with phosphate-buffered saline (PBS) and ECs were grown for 6-72 h in supplemented EBM-2 MV with 5% FBS and VeroE6 in DMEM with 8% FBS. Infected cells were detected using anti-Nucleocapsid (40588-T62, Sino Biological) and immunoperoxidase staining with (HRP)-labeled anti-mouse IgG (1:2,000; KPL-074-1806), and 3-amino-9-ethylcarbazole. Lentivirus-transduced hPMECs and hBMECs expressing human ACE2 (rACE2-hPMEC/rACE2-hBMEC) were similarly infected as described above for 2, 6, 12, 24, 48 and 72h. SARS-CoV-2 titers were determined by serial dilution on VeroE6 cells, quantifying SARS-CoV-2 infected cell foci 16 hpi by immunoperoxidase staining.

**qRT-PCR analysis.** hPMEC, hBMEC, hCMEC, hGMEC, HUVEC, HEK293T, VeroE6 and Calu-3 cells were lysed in RLT buffer, and total RNA was purified on RNeasy columns (Qiagen). cDNA synthesis was performed using a Transcriptor first-strand cDNA synthesis kit (Roche) using random hexamers as primers (25°C for 10 min, 50°C for 60 min, 85°C for 5 min). qRT-PCR primers for ACE2: forward, GACAAGAGCAAACGGTTGAACAC; reverse, GCCCAGAGCCTCTCATTGTAG. Genes were analyzed using PerfeCTa SYBR green SuperMix (Quanta Biosciences) on an CFX96 Real-Time PCR system (Bio-Rad). Internal glyceraldehyde-3-phosphate dehydrogenase (GAPDH) mRNA levels were used for normalization and the fold expression was calculated using the 2^−ΔΔCT^ method compared to the HEK293T RNA levels. Alternatively, rACE2-hPMEC were infected with SARS-CoV-2 (MOI, 1) for 6, 12 and 24 h, RNA was isolated and cDNA synthesis was performed as described above. qRT-PCR primers for specific genes were designed according to the NCBI gene database with 60°C annealing profiles and responses were calculated using the 2^−ΔΔCT^ method compared to the hPMEC RNA levels.

**Western blotting.** Mock or SARS-CoV-2 infected hPMEC, hBMEC, hCMEC, hGMEC, HUVEC, HEK293T, VeroE6 and Calu-3 cells were harvested 24 hpi, PBS washed and subsequently lysed in buffer containing 1% NP-40 and 0.1% SDS (150 mM NaCl, 50 mM Tris-Cl, 10% glycerol, 2 mM EDTA, 10 nM sodium fluoride, 2.5 mM sodium pyrophosphate, 2 mM sodium orthovanadate, 10 mM -glycerophosphate) with protease inhibitor cocktail (Sigma). Total protein levels were determined in a bicinchoninic acid assay (Thermo Scientific), and 10 µg of protein was resolved by 4-15% SDS polyacrylamide gel electrophoresis. Proteins were transferred to nitrocellulose, blocked in 5% bovine serum albumin (BSA), and incubated with anti-ACE2 antibody (10108-T24, Sino Biological) or anti-GAPDH (G9545; Sigma-Aldrich). Protein was detected using horseradish peroxidase (HRP)-conjugated anti-rabbit secondary antibody (Amersham) and Luminata Forte Western HRP substrate (Millipore).

**Confocal immunofluorescence.** SARS-CoV-2 (MOI, 1) was adsorbed to 90% confluent rACE2-hPMEC monolayers for 1 h in Lab-Tek II chambers (Nunc), washed with PBS, and grown in supplemented EBM-2 MV with 5% FBS. At the indicated times, cells were washed with PBS and fixed for 10 min with cold 100% methanol. Cells were blocked using 5% BSA in PBS for 2 h and incubated with a combination of antibody pairs: anti-Nucleocapsid mouse monoclonal (1:100; 40143-MM08, Sino Biological) and anti-ACE2 rabbit polyclonal (1:2,000; 10108-T24, Sino Biological); anti-Nucleocapsid rabbit polyclonal (1:4,000) and anti-PECAM-1 mouse monoclonal (1:100; 89C2, Cell Signaling); anti-Nucleocapsid rabbit polyclonal (1:4,000) and anti-Thrombomodulin mouse monoclonal (1:100; MA5-11454, Invitrogen), diluted in blocking solution for 18 h at 4°C. Cells were washed and incubated for 2 h with Alexa 488-conjugated goat anti-mouse IgG antibody (Invitrogen) and with Alexa 546-conjugated goat anti-rabbit IgG antibody (Invitrogen) diluted 1:700 in blocking solution at room temperature. Cells were subsequently incubated with 5 µM 4,6-diamidino-2-phenylindole (DAPI; Sigma) for 5 min at room temperature. Slides were mounted using ProLong Antifade (Thermo Fisher) solution and observed using a Zeiss LSM 510 META/NLO confocal microscope.

**Statistical analysis.** Results shown in each figure were derived from two to three independent experiments with comparable findings; the data presented are means ± standard errors of the means (SEM), with the indicated P values of 0.05, 0.01, and 0.001 considered significant. One-way comparisons were performed using analysis of variance (ANOVA) with Turkey’s post-hoc test. All analyses were performed using GraphPad Prism software version 4.0.
